# Translating discoveries as novel biomarkers and interventions in ophthalmology

**DOI:** 10.1186/s12967-023-04216-1

**Published:** 2023-06-13

**Authors:** Geoffrey K. Broadhead, Levon M. Khachigian

**Affiliations:** 1 Save Sight Institute, University of Sydney, Sydney, NSW 2006 Australia; 2 School of Biomedical Sciences, Faculty of Medicine and Health, University of New South Wales, UNSW, Sydney, 2031 Australia

We are delighted to announce the launch of a new section in the *Journal of Translational Medicine* entitled “Translational Ophthalmology”. This section is focused on the rapidly expanding field of translational medicine as it relates to ophthalmology and vision science, as well as the role of ophthalmology in guiding the diagnosis and care of other (non-ophthalmic) diseases. *Translational Ophthalmology* is a section primarily aimed at (but not limited to) studies focused on mechanisms and development of novel therapies for ophthalmic conditions, new biomarkers of ocular pathology, and the role of ophthalmology and ophthalmic findings in the diagnosis and management of systemic medical conditions.

There are a number of factors that make ophthalmology and visual science unique compared to other areas of medicine, and that present both specific challenges and opportunities for ophthalmic care. While the immune-privileged nature of the eye can intrinsically protect the eye from inflammatory insults*,* this can limit the absorption and efficacy of systemically administered medications and reduce the risk of ocular side effects of agents administered systemically. Hence, local routes of administration, such as intravitreal injection, to treat ocular diseases will likely remain standard-of-care for certain agents [1, 2]. Utilization of novel therapeutics in this setting requires an understanding of specific intraocular pharmacokinetics and pharmacodynamics, and that of delivery modalities and their properties [2]. Similarly, the complex nature of ocular tissue, which consists of a combination of cell types including both CNS neurons and ophthalmic-specific cells, means that therapies developed for ophthalmic conditions may need to consider potential effects on different components of the eye [1–3].

The relative ease of sampling certain ophthalmic fluids, particularly tears, but also to some extent aqueous or vitreous humor, has allowed for extensive investigation of the ocular biome [4, 5]. This has permitted the development of a number of potential biomarkers of ophthalmic diseases, including dry eye and diabetic retinopathy [4, 5]. However, acquiring ophthalmic tissue without affecting the integrity of the eye can be more complex than for some other organ systems, and this can present unique challenges in investigating ophthalmic diseases and their molecular pathogenesis.

The ease with which most ophthalmic tissues can be imaged and studied has also allowed for the development of a range of biomarkers based on clinical investigation results, rather than just laboratory findings. This has resulted in the development of several anatomical and functional investigations of the eye and visual disorders, including optical coherence tomography (OCT) and OCT-angiography, automated visual fields and electroretinography, all of which can be conducted in a repeatable and non-invasive fashion [6, 7]. There is a growing appreciation of the value of these technologies as both markers of disease activity and measures of response to treatment, not just for ophthalmic disease but also disorders affecting other organ systems. There is a growing tide of novel therapeutic approaches in development for ocular diseases [8].

The connection between the eye and the CNS, coupled with the relative ease with which the eye may be studied using lab-based approaches and clinical biomarkers, has led investigators to examine ocular characteristics as indicators of systemic diseases, specifically neurological disorders. To this end, there is a growing field of research on the role of ophthalmic findings as potential biomarkers for Multiple Sclerosis, Parkinson’s disease and Alzheimer’s Disease, amongst others [9, 10]. This avenue provides the exciting possibility of utilizing the low-risk and repeatable nature of most ophthalmic test findings as a window into diseases affecting other organ systems without the end for more complex or involved systemic investigations.

This new section within the *Journal of Translational Medicine* provides a platform for research into the expanding field of translational ophthalmology and will facilitate the publication and discussion of high-quality peer-reviewed studies in this area. This new section of the journal is keen to receive your submissions!
